# Changes in substance use and engagement in gaming/gambling in persons with severe mental illness during the COVID-19 pandemic and earthquakes: a community study in two points

**DOI:** 10.3389/fpsyt.2023.1264875

**Published:** 2023-12-15

**Authors:** Sara Medved, Irena Rojnić Palavra, Josefina Gerlach, Sarah Levaj, Laura Shields-Zeeman, Felix Bolinski, Zoran Bradaš, Zoran Madžarac, Igor Filipčić, Martina Rojnić Kuzman

**Affiliations:** ^1^Department of Psychiatry and Psychological Medicine, University Hospital Centre Zagreb, Zagreb, Croatia; ^2^University Psychiatric Hospital Sveti Ivan, Zagreb, Croatia; ^3^Netherlands Institute of Mental Health and Addiction (Trimbos Institute), Utrecht, Netherlands; ^4^Faculty of Dental Medicine and Health, Josip Juraj Strossmayer University of Osijek, Osijek, Croatia; ^5^School of Medicine, University of Zagreb, Zagreb, Croatia

**Keywords:** addiction, COVID-19 pandemic, severe mental illness, earthquake, community mental health team, (CMHT)

## Abstract

**Introduction:**

External stressors, such as COVID-19 pandemic and earthquake, can cause an increase in substance use and addictive behavior in persons with severe mental illnesses (SMI). We analyzed the changes and predictors of substance use and addictive behavior in SMI during these double disasters in Croatia.

**Methods:**

Questionnaires exploring the presence of substance or behavior addiction disorder, mental ill health [Depression Anxiety Stress Scales-21 (DASS-21), Insomnia Severity Index (ISI), Perceived Stress Scale (PSS), Obsessive-Compulsive Inventory-Revised], coping mechanisms, and perceived social support [Multidimensional Scale of Perceived Social Support (MSPSS)] were administered among 90 participants with SMI included in the RECOVER-E study in May/June 2020 (first COVID-19 wave, Zagreb earthquake) and in December 2020/January 2021 (second COVID-19 wave, Petrinja earthquake).

**Results:**

In both time points, a major increase was observed in tobacco smoking (25.0%; 28.6%, respectively) predicted by discontinuation of antidepressants and higher DASS-21 score. Increased sedative use was observed (24.4%; 23.8%, respectively) predicted by higher PSS and ISI scores, lower MSPSS scores, antipsychotic discontinuation and not receiving community mental health team (CMHT) service.

**Discussion:**

In persons with SMI during a double disaster special attention needs to be given to reducing mental-ill health and stress, providing social support and continuity of psychiatric care, through medications and CMHTs.

## 1 Introduction

The COVID-19 pandemic has necessitated the implementation of preventive measures, which have had a detrimental impact on mental health (1–3). Additionally, psychiatric services have faced significant reductions (4). Persons with severe mental illnesses (SMI) have shown to be highly sensitive to the changes brought by the COVID-19 pandemic (5).

Unfortunately, Croatia experienced two major earthquakes during the pandemic. On March 22nd, 2020, the capital of Croatia, Zagreb was struck by an earthquake measuring 5.5 on the Richter scale, causing extensive damage to numerous healthcare facilities (6). On December 29th, another earthquake measuring 6.4 on the Richter scale occurred near Zagreb, specifically in Petrinja, which is approximately 40 kilometers away (7). The impact of earthquake can exacerbate addictive behavior and influence the factors related to mental ill-health and the utilization of mental health services (8–10). Double disasters, such as the pandemic and earthquakes in this case, pose a unique challenge to the mental health of populations (11, 12). Globally, changing conditions directly affect the complexity of current and future disaster management issues (11). Previous research has underscored the necessity for special attention and long-term support in addressing the psychological impacts of double disasters (12, 13). Higher incidence of posttraumatic stress disorder, depression, and anxiety have been reported following such disasters (12, 13). Certain pre-existing psychiatric conditions, such as obsessive-compulsive disorder, may exhibit significant clinical worsening (14). Experiencing loss, displacement from one’s place of residence, socio-economic challenges, lack of social support, and negative coping mechanisms, such as substance abuse, can induce psychological stress (13). Moreover, persons with SMI are particularly prone to experiencing higher levels of stress compared to the general population (15). They also inherently express a higher rate of certain psychoactive substances and behavioral addictive disorders (16). Discontinuation of pharmacological therapy may further exacerbate their condition (13). Therefore, the occurrence of a double disaster can potentially place persons with mental illnesses in an extremely vulnerable position.

The primary aim of this research is to examine changes in substance use and addictive behavior in persons with SMI during the first and second double disasters—the COVID-19 waves and the co-occurring earthquakes. Furthermore, we seek to explore mental ill-health, coping mechanisms, psychiatric treatment, and perceived social support as predictors of changes in substance use and addictive behavior.

## 2 Materials and methods

### 2.1 Study design

This research was conducted at the Department of Psychiatry and Psychological Medicine, University Hospital Centre (UHC) Zagreb, as part of the RECOVER-E project (Large-scale implementation of community based mental health care for people with severe and enduring mental ill health in Europe) (17, 18). The primary aim of the RECOVER-E study is to implement and evaluate a model of a community-based mental health service, community mental health teams (CMHTs), to people with SMI and compare it to the treatment as usual (TAU) at five different sites, including UHC Zagreb. The participants for RECOVER-E were consecutively recruited from 2018 at the UHC Zagreb if they were adults diagnosed with SMI (schizophrenia and other psychotic disorders, bipolar-affective disorder, or major depressive disorder) according to ICD-10 (International Classification of Diseases 10th Revision) and randomized in the group receiving CMHT or TAU. More details of the RECOVER-E study aims and design can be found elsewhere (17). RECOVER-E and this extension of the research were approved by the Ethics Committee of UHC Zagreb (class: 8.1-18/149-2, number: 02/21 AG).

The first measurement in this particular extension of the study was conducted from May to June 2020; in the midst of the first pandemic wave (19), and shortly after the Zagreb earthquake, during which a very stringent set of restrictions was introduced (20, 21). Inpatient treatment was provided only for emergency conditions (22, 23), and other services were transferred to telepsychiatry.

The second measurement in this extension was conducted during the second pandemic wave from December 2020 to January 2021 (19), and shortly after the Petrinja earthquake. At that point, mass vaccination program had started, and a soft lockdown was introduced (21). However, the overall health system was under much higher pressure compared to the first wave (20, 21). The acute psychiatric inpatient unit at UHC Zagreb was repurposed to COVID-19 ward, so all patients requiring hospitalization were transferred to another inpatient facility. Day hospitals resumed their work, whereas outpatient care continued with telepsychiatry and reduced in-person visits.

### 2.2 Participants

In May 2020, all RECOVER-E project participants in CMHT and TAU group were contacted and asked to engage in this additional research. Upon signing the informed consent, survey and questionnaires were administered by telephone. Participants could withdraw from the study at any time without any consequences on their participation in the main project. From March 2020 until the end of the project (February 2021), CMHT home visits were transformed to continuous telepsychiatry (online and telephone services) with occasional in-person interventions.

### 2.3 Materials

Survey and questionnaires were used for the assessment, which took up to 45 min for the completion. All the materials, except for the coping mechanism evaluation were applied in both time points. The materials were collected over the telephone by independent investigators (SM, JG, and SL) not involved in providing either CMHT or TAU. The investigators have been trained in applying questionnaires and collecting the data via telephone. During the study, the investigators were having regular meetings and consensually agreed on all questionnaires applied in the research.

#### 2.3.1 Survey measures

The survey collected socio-demographics and medical information. Socio-demographic component collected the data on age, sex, marital status, employment, education, and household. Information about psychiatric diagnosis, received service (TAU or CMHT), and psychiatric medication was obtained. Self-reported changes in substance use and addictive behavior was assessed with response categories “no consumption,” “no changes in use,” “increased use” or “decreased use” for use of alcohol, tobacco smoking, cannabis use, sedative use, and other drugs. Gaming and gambling use was assessed with response categories “never,” “no changes in use,” “more often use” or “less often.”

##### 2.3.1.1 Questionnaires

Questionnaires were used to assess the presence of addiction disorders, mental ill-health, coping mechanisms and perceived social support. All questionnaires were applied in Croatian language, using the validated versions of the questionnaires in Croatian population, apart from OCI-R and DASS-21, which were not previously validated. These two questionnaires were translated and back-translated into Croatian language by an English and Croatian native speaker.

The presence of alcohol, drug and gambling addiction disorders were assessed using standardized scales:

1. Alcohol Use Disorders Identification Test (AUDIT) is a 10-item questionnaire providing data on alcohol consumption, drinking behavior, and alcohol-related problems. A range from 1 to 7 suggests low-risk consumption; from 8 to 14 hazardous or harmful alcohol consumption and a score from 15 or more indicates the likelihood of alcohol dependence (24).
2. Drug Use Disorders Identification Test (DUDIT) is an 11-item self-administered screening instrument for substance abuse/harmful use and dependence according. If a male patient shows a score of 6 or more, or a female patients a score of 2 or more, he or she probably has drug related problems – either substance abuse/harmful use or dependence. If a patient (both genders) shows a score of 25 points or more, it is highly probable that he or she is dependent on one or more drugs (25).
3. The South Oaks Gambling Screen (SOGS) is a 20-item multiple-choice instrument that was introduced for identifying individuals with pathological gambling. Positive responses to 5 or more items indicate a “probable pathological gambler” (26).

Mental ill-health was assessed using questionnaires for exploring symptoms of depression, anxiety, stress, insomnia, and exacerbation of compulsive obsessive symptoms:

1. Depression Anxiety Stress Scales-21 (DASS-21) rates symptoms of depression, anxiety, and stress through 21 items rated from 0 (did not apply to me at all) to 3 (applied to me almost completely or most of the time). The depression subscale contains items related to hopelessness, depressed mood, feeling worthlessness of life, lack of interest and involvement in daily activities, anhedonia, and ideas of guilt. Anxiety is assessed by items about physical changes in the body, anxiety related to different life situations and subjective experience of fear. Stress is assessed by items about tension, irritability, and overreaction. The final score for each subscale provides four severity ranges: mild, moderate, severe, and extremely severe (27).
2. Insomnia Severity Index (ISI) is a seven-item questionnaire that assesses the quality of sleep in the past 2 weeks using a 5-point Likert scale (0–4). The following dimensions are evaluated: difficulties falling asleep, sleep maintenance, early morning awakening problems, sleep dissatisfaction, interference of sleep difficulties with daytime functioning and quality of life and distress caused by the sleep difficulties. The total score ranges from 0 to 28, and higher scores indicate greater sleep difficulties (28).
3. Perceived Stress Scale (PSS) is used to measure the degree to which situations in one’s life are appraised as stressful. It consists of 10 items, using Likert’s s Scale for scoring. The total score ranges from 0 to 40 with higher scores indicating higher perceived stress (29).
4. Obsessive-Compulsive Inventory – Revised (OCI-R) which is an 18-item instrument that uses the Likert scale (0–5) to assess experiences in everyday life that belong to spectrum of obsessive-compulsive disorder. The score range is between 0 and 72 (30).
5. Coping mechanisms were assessed using Brief Resilient Coping Scale (BRCS). BRCS quantifies the ability to recover from a stressful situation with respect to behavior and activities of the individual using a Likert scale (1**–**5). The total score ranges from 4 to 20 with higher score indicating high resilient coping (31).
6. Finally, the Multidimensional Scale of Perceived Social Support (MSPSS), a 12-item scale was used to measure perceived social support from three sources: family, friends, and a significant other on a scale from 1 (I do not agree at all) to 7 (I completely agree). The mean score for each subscale is calculated by summing across items from that subscale and then dividing by 4, with the score range from 1 to 7 (32).

All questionnaires were selected based on their good validity and reliability. We used the Croatian versions of AUDIT, DUDIT, SOGS, DASS-21, ISI, OCI-R, MSPSS and the English versions of PSS and BRCS.

#### 2.3.2 Study outcomes

The primary outcome was the evaluation of changes in substance use and addictive behavior in persons with SMI in the first and second study point. The secondary outcome was the analysis of predictors of increased substance use and addictive behavior. The outcomes in the first and second time point were not compared directly, due to different circumstances that could have impacted outcomes of this study.

#### 2.3.3 Statistical analysis

We calculated the required sample size for secondary outcomes with a targeted statistical power of 0.80, a significance level of *p* < 0.05, two tailed, for logistic regression, using the means and SD of quantitative variables (DASS) and the minimum odds ratio of 1.1. Under these conditions we needed up to 80 respondents. Anticipating around 10% of the data would be incorrectly collected, we estimated the initially required sample size to 90 participants. We calculated the required sample size using the G*Power version 3.1.9.4 (33). Descriptive analysis was used for sample description. For the primary outcome analysis, variables describing the changes in the use of substances and addictive behavior were recoded into binary variables “increased use” and “other” that combined those stable or with decreased use or addictive behavior due to the small sample size in one of the groups. Odds ratios were estimated through binary logistic regression to predict the increased use of these dependent variables by sex, age and variables indicating mental ill-health (PSP, OCI-R, ISI, DASS-21), coping styles (BRCS), support system (MSPSS), received psychiatric service (TAU or CMHT group), and psychiatric medications. The results were interpreted at the 5% significance level (α = 0.05). The statistical program STATA/IC 15.1 Stata Corp LLC was used for statistical analysis. We used the False discovery rate (FDR) set at 5% to control the effect of multiple testing considering the primary and secondary outcomes testing (34).

## 3 Results

**Figure 1:**
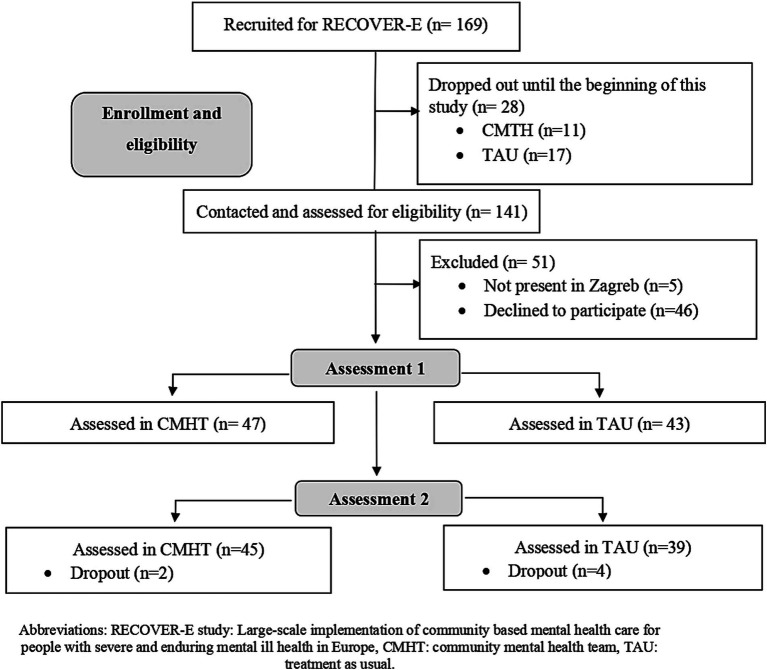
Flow chart of analysis sample.

The sampling is shown in Figure 1. From overall 169 RECOVER-E participants, 90 participants engaged in the first measurement of this study. The assessment included 47 participants included in the CMHT group and 43 in TAU group. Altogether six participants dropped out in the second measurement: two in the CHMT group and 4 in the TAU group. There was no statistically significant difference between the control and intervention group (TAU vs. CMHT group) in socio-demographic and medical data, except in the use of long acting injectables (LAI) (CMHT group received LAIs more than TAU group). We refer the reader to another publication with more details about the population (35).

Table 1 shows baseline socio-demographic and medical data of the population and scores of questionnaires exploring the presence of substance or behavior addiction disorder, mental ill-health, coping mechanisms, and perceived social support in the first and second study point.

**Table 1:** Baseline sociodemographic and medical data of participants (*N* = 90)*.

Variable	*N* (%)^*^
**Socio-demographic characteristics**	
Male	39 (43.3)
Age (years), mean (SD)^*^	41.9 (14.6)
Single/divorced	62 (68.9)
Employed	20 (22.7)
Finished high school	79 (90.8)
Living alone	12 (13.3)
Mean number of persons in household (SD)	2.7 (1.3)
Mean number of children in household (SD)	0.3 (0.7)
**Psychiatric diagnosis**	
Schizophrenia and other psychotic disorders	63 (70.0)
Major depressive disorder	19 (21.1)
Bipolar-affective disorder	8 (8.9)
**Provided treatment**	
CMHT	47 (52.2)
TAU	43 (47.8)
**Psychiatric medication**	
Oral antipsychotics	79 (88.8)
LAIs	23 (27.4)
Mood stabilizers	26 (29.2)
Antidepressants	33 (37.1)
Sedatives	56 (62.9)

Questionnaires overall scores		
Mean score (SD)^*^	1st assessment	2nd assessment (*N* = 84)
DASS-21	37.4 (32.8)	35.6 (30.6)
ISI	7.8 (6.4)	9.7 (6.2)
PSS	20.0 (6.0)	20.6 (5.2)
OCI-R	9.1 (10.3)	9.6 (10.1)
BRCS	12.6 (3.6)	–
Low coping (*N* (%))	43 (48.9)	–
Moderate coping (*N* (%))	39 (44.3)	–
High coping (*N* (%))	6 (6.7)	–
MSPSS significant others	5.1 (2.2)	5.4 (2.2)
MSPSS friends	4.8 (2.1)	4.9 (2.2)
MSPSS family	5.8 (1.6)	5.9 (1.6)
**MSPSS**		
High social support (*N* (%))	49 (56.9)	57 (69.5)
Moderate social support (*N* (%))	32 (37.2)	22 (26.8)
Limited social support (*N* (%))	5 (5.8)	3 (3.7)

^*^Unless otherwise specified. SD, standard deviation; CMHT, community mental health team; TAU, treatment as usual; LAI, long acting injectables; DASS-21, Depression Anxiety Stress Scales-21; ISI, Insomnia Severity Index; PSS, Perceived Stress Scale; OCI-R, Obsessive-Compulsive Inventory - Revised; BRCS, Brief Resilient Coping Scale; MSPSS, Multidimensional Scale of Perceived Social Support.

Within the sample, approximately 2.4% of participants (*N* = 2) were identified with harmful alcohol consumption and 3.4% (*N* = 3) with alcohol dependence using AUDIT, 1.2% (*N* = 1) with probable drug abuse using DUDIT and 5.8% (*N* = 5) with probable gambling problem using SOGS in the first assessment. In the second assessment, approximately 2.4% (*N* = 2) were showing harmful use of alcohol and 1.2% (*N* = 1) of participants were identified as alcohol dependent, 1.2% (*N* = 1) were having probable drug abuse, while nobody identified as a pathological gambler. Since increased alcohol consumption, psychoactive substance use, gaming, and gambling measured by AUDIT, DUDIT and SOGS were reported by less than 5 persons, we did not include it in the further analyses.

The major increase was observed in the use of tobacco smoking and sedatives (Table 2). All data about self-reported changes in substance use and addictive behavior are presented in Table 2.

**Table 2:** Assessment of self-reported changes of substance use and addictive behavior.

Self-reported substance use and addictive behavior	1st assessment (*N* = 90), *N* (%)	2nd assessment (*N* = 84), *N* (%)
**Alcohol**		
Do not use	75 (89.3)	76 (90.5)
No change	3 (3.6)	2 (2.4)
Increased	2 (2.4)	3 (3.6)
Decreased	4 (4.8)	3 (3.6)
**Tobacco smoking**		
Do not smoke	42 (50.0)	43 (51.2)
No change	13 (15.5)	9 (10.7)
Increased	21 (25.0)	24 (28.6)
Decreased	8 (9.5)	8 (9.5)
**Cannabis**		
Do not use	83 (92.2)	84 (100.0)
No change	6 (6.7)	0 (0.0)
Increased	0 (0.0)	0 (0.0)
Decreased	1 (1.1)	0 (0.0)
**Drugs**		
Do not use	83 (92.2)	83 (98.8)
No change	7 (7.8)	1 (1.2)
Increased	0 (0.0)	0 (0.0)
Decreased	0 (0.0)	0 (0.0)
**Sedatives**		
Do not use	34 (41.5)	50 (59.5)
No change	24 (29.3)	7 (8.3)
Increased	20 (24.4)	20 (23.8)
Decreased	4 (4.9)	7 (8.3)
**Gaming**		
Never	71 (78.9)	75 (89.3)
No change	9 (10.0)	2 (2.4)
More	7 (7.8)	7 (8.3)
Less	3 (3.3)	0 (0.0)
**Gambling**		
Never	81 (90.0)	75 (89.3)
No change	8 (8.9)	3 (3.6)
More	0 (0.0)	5 (5.9)
Less	1 (1.1)	1 (1.2)

A higher DASS-21 score was a positive predictor of increased tobacco use in the second assessment (*p =* 0.021, OR 1.102, 95% CI 1.015–1.197). In the first assessment, increased sedative use was predicted in participants not receiving CMTH service, i.e., those in TAU group (*p =* 0.031, OR 8.225, 95% CI 1.208–56.015), by discontinuation in antipsychotic medications (*p* = 0.010, OR 0.020, 95% CI 0.001–0.386) and by higher PSS score (*p* = 0.011, OR 1.216, 95% CI 1.046–1.413). Higher ISI and MSPSS family subscale scores predicted higher sedative use in the second assessment (*p =* 0.007, OR 1.222, 95% CI 1.057–1.413; *p =* 0.034, OR 0.576, 95% 0.346–0.959, respectively). All results remained statistically significant even after adjustment with the FDR set at 5%. Details are shown in Table 3.

**Table 3:** Predictors of the increase smoking and sedative use in participants.

Variable	Increased tobacco smoking					
	1st assessment (*N* = 85)			2nd assessment (*N* = 79)		
	*p*	OR	95% CI	*p*	OR	95% CI
Sex (female)	0.462	1.619	0.449–5.843	0.675	1.436	0.265–7.792
Age	0.573	1.013	0.968–1.060	0.700	0.989	0.935–1.046
No CMHT	0.225	0.451	0.124–1.633	0.236	0.372	0.073–1.906
Antipsychotics	0.978	1.027	0.151–7.006	0.342	0.380	0.051–2.802
LAI	0.459	0.597	0.153–2.335	0.222	0.329	0.055–1.960
Mood stabilizers	0.669	1.309	0.380–4.513	0.114	4.182	0.708–24.712
Antidepressants	0.964	0.971	0.274–3.442	**0.046**	**0.152**	**0.024–0.966**
Benzodiazepines	0.755	0.816	0.227–2.938	0.428	0.514	0.099–2.662
ISI	0.824	0.979	0.812–1.180	0.684	1.029	0.898–1.178
PSS	0.342	1.054	0.946–1.173	0.451	1.082	0.881–1.329
OCI	0.437	1.057	0.919–1.215	0.847	0.992	0.918–1.073
MSPSS others	0.538	1.019	0.959–1.083	0.283	0.750	0.443–1.269
MSPSS friends	0.812	1.008	0.942–1.080	0.270	1.398	0.770–2.537
MSPSS family	0.462	1.148	0.795–1.658	0.311	1.317	0.773–2.246
DASS-21	0.436	1.167	0.791–1.723	**0.021**	**1.102**	**1.015–1.197**
BRCS	0.787	1.063	0.684–1.651	0.323	0.890	0.706–1.122

Variable	Increased sedatives use					
	1st assessment			2nd assessment		
	*p*	OR	95% CI	*p*	OR	95% CI
Sex (female)	0.344	0.409	0.064–2.608	0.837	0.828	0.137–5.005
Age	0.323	0.971	0.915–1.030	0.543	1.018	0.961–1.079
No CMHT	**0.031**	**8.225**	**1.208–56.015**	0.137	4.170	0.634–27.444
Antipsychotics	**0.010**	**0.020**	**0.001–0.386**	0.601	2.489	0.081–76.194
LAI	0.066	6.227	0.887–43.696	0.692	0.669	0.092–4.879
Mood stabilizers	0.255	0.304	0.039–2.368	0.712	1.396	0.238–8.206
Antidepressants	0.839	0.839	0.154–4.568	0.903	1.109	0.211–5.835
Benzodiazepines	0.878	1.167	0.163–8.371	0.971	0.966	0.150–6.215
ISI	0.844	1.029	0.777–1.362	**0.007**	**1.222**	**1.057–1.413**
PSS	**0.011**	**1.216**	**1.046–1.413**	0.814	0.979	0.819–1.170
OCI	0.704	0.963	0.792–1.171	0.865	1.007	0.927–1.094
MSPSS others	0.119	1.076	0.981–1.181	0.076	1.940	0.933–4.032
MSPSS friends	0.222	0.940	0.851–1.038	0.145	0.646	0.359–1.163
MSPSS family	0.426	1.215	0.753–1.960	**0.034**	**0.576**	**0.346–0.959**
DASS-21	0.416	1.242	0.737–2.091	0.831	1.008	0.936–1.086
BRCS	0.833	1.062	0.609–1.850	0.215	0.870	0.698–1.084

OR, odds ratio; CI, confidence interval; CMHT, community mental health team; LAI, long acting injectables; DASS-21, Depression Anxiety Stress Scales-21; ISI, Insomnia Severity Index; PSS, Perceived Stress Scale; OCI-R, Obsessive-Compulsive Inventory - Revised; BRCS, Brief Resilient Coping Scale; MSPSS, Multidimensional Scale of Perceived Social Support. Bold values indicate statistically significant value (*p* < 0.05).

## 4 Discussion

The results of our study revealed a noteworthy increase in tobacco smoking and the use of sedatives among individuals with SMI during both instances of the double disaster. It is important to note that the frequency of psychoactive substance use in this sample was notably lower than what has been reported in previous literature (36). This disparity can be attributed to several factors, including the implementation of anti-COVID-19 measures, such as the “stay-at-home” national policy, which disrupted conventional sources of drugs and led to an observed increase in pricing (37). Drug availability is one of the important risk factors for addiction (38). Additionally, specific cultural standards regarding addiction within the socio-cultural model may have biased self-reporting (39).

Despite the relatively small number of individuals exhibiting harmful use and behavior in our sample, a significant increase in smoking and sedative use was evident at both study points, mirroring trends in the general population (40). Notably, a higher prevalence of tobacco smoking was already documented among individuals with SMI in the pre-pandemic time (41), amplifying their health burden (42). The increase in sedative use is also a cause for concern, given its association with a higher risk of mortality (43).

The use of tobacco was predicted by depression and anxiety symptoms and discontinuation of antidepressant use, whereas increased use of sedatives was predicted by insomnia, not- receiving CMHT treatment, discontinuation of antipsychotics, and perceived levels of stress and social support. High environmentally – induced stress contributes to mental-ill health and elicits different, sometimes maladaptive coping responses, including the use of substances (44). It is worth noting that environmental stressors, which can contribute to mental ill-health and maladaptive coping responses, including substance use, played a significant role in the context of the COVID-19 pandemic and earthquakes (44, 45). The documented increase in depression, anxiety, and stress symptoms during these challenging times aligns with findings from other studies (46–48). Importantly, depression and anxiety symptoms have known associations with addictive behavior (46), while stress is independently associated with SMI (49). This heightened stress level not only exacerbates the risk of substance use and addictive behavior but also underscores the importance of effective mental health interventions, particularly in times of crisis.

Our study found that participants who reported receiving greater social support were at a lower risk of engaging in substance use. This finding aligns with previous research that has shown a significant relationship between the lack of social support and an increased risk of addiction during the pandemic (36). Social support plays a crucial role in promoting recovery (50) and can act as a protective factor against the negative impact of stigma and shame. This, in turn, has a positive effect on an individual’s quality of life and mental health (51).

Psychiatric treatment has been shown to have a significant effect on tobacco and sedative use. Participants receiving CMHT treatment had a lower risk for substance use. CMHTs as an outreach service have been recognized as a valuable resource for persons with SMI and addictive disorders for many years (52). The availability of mental health services, such as CMHTs, has been shown to have multiple benefits. It can enhance treatment adherence, alleviate anxiety associated with the loss of service support, and provide essential medical advice (53). Notably, in some situations, CMHTs were the only accessible psychiatric service for individuals with SMI during the pandemic and earthquakes in Croatia (10). This highlights their pivotal role in ensuring continued care for the most severely affected patients, presenting a novel approach to healthcare during crises (54, 55). Furthermore, it is recommended that CMHTs should not be limited to pandemic times but should be sustained and expanded beyond such emergencies (56, 57). This model of care should be developed and implemented in countries where it does not currently exist (56, 57). There is a scarcity of studies examining the impact of the organization of psychiatric services, including CMHT services, on individuals with SMI during a pandemic or natural catastrophe. While a few examples exist (11, 12, 35, 58), further research in this area is warranted to better understand their role and effectiveness in crisis situations.

The discontinuation of antidepressant medications emerged as a predictor of increased tobacco use in our study. This finding is noteworthy considering that effective treatments for smoking cessation are currently available (59), and efforts to raise awareness about cessation strategies during the pandemic have been initiated (60). These results may indicate a potential gap in healthcare. Similarly, the increase in sedative use was predicted by the discontinuation of antipsychotic treatment. This could be explained by the shortfall of sleep induction (61). The discontinuation of psychiatric treatment is attributable to the lock-down measures and the disruption of the standard care in the assessment (35).

These results highlight the profound impact of external stressors on the unhealthy lifestyles of individuals with SMI. As mentioned earlier, individuals with SMI are inherently vulnerable, and additional burdens can significantly compromise their well-being (42, 43). Unfortunately, the anticipated rise in the frequency of natural disasters in the coming years (62) necessitates proactive measures.

During and after a natural disaster, the mental and physical health of marginalized populations, including those with SMI, is particularly at risk (63). Clinicians must be well-prepared and equipped with the skills and knowledge required to deliver continuous psychiatric care to individuals with SMI, especially in cases of double disasters. This includes the capacity to provide mental health services in the community, tailored to the specific needs of the SMI population, and in coordination with other essential public services, such as social services, labor services, and housing. Additionally, policymakers must prioritize emergency preparedness and response strategies tailored to the needs of vulnerable populations during crises (63). Given the far-reaching impacts of natural disasters, cross-national responses may be necessary. This was especially evident during the pandemic, underlining the crucial role played by international associations and informal organizations in alleviating the effects of traumatic events. They have the potential to develop scientifically universal guidelines and algorithms specifically designed for persons with SMI, applicable to various domestic circumstances at every level. With well-structured preventive measures in place for persons with SMI, the risks to their well-being can be significantly mitigated.

### 4.1 Limitations

First, many of the measures used in this study are based on self-report, as there is a lack of more objective measures for assessing the true consumption of substances. Secondly, the absence of research data collected before the outbreak of the pandemic and earthquakes, using the same standardized scales, prevents us from confirming that the observed results are a direct consequence of the COVID-19 pandemic and/or earthquakes. Other unmeasured factors may have influenced the outcomes. Furthermore, the cross-sectional design of the study limits our ability to establish causality between the pandemic, earthquakes, and substance abuse/addictive behavior. The study also does not address the longer-term effects of the COVID-19 pandemic. Some effects of the disruption of mental health care and the impact of stressors may become more apparent after a more extended period. Lastly, the sample size of participants in this study was relatively small, which may limit the ability to perform advanced statistical analyses or draw generalizable conclusions. Future research with larger sample sizes would be beneficial in confirming and extending these findings.

## 5 Conclusion

In conclusion, in a case of a double disaster, additional health burden due to tobacco smoking and sedative use in persons with SMI needs to be foreseen. To counteract the effect, special attention needs to be given to reducing mental-ill health and stress, providing social support and continuity of psychiatric care, through both medications and CMHTs.

## Data availability statement

The raw data supporting the conclusions of this article will be made available by the authors, without undue reservation.

## Ethics statement

The studies involving humans were approved by the Ethics Committee of University Hospital Centre Zagreb. The studies were conducted in accordance with the local legislation and institutional requirements. The participants provided their written informed consent to participate in this study.

## Author contributions

SM: Conceptualization, Data curation, Formal analysis, Investigation, Methodology, Writing – review & editing, Writing – original draft. IRP: Supervision, Writing – review & editing. JG: Investigation, Writing – original draft, Project administration, Writing – review & editing. SL: Conceptualization, Data curation, Investigation, Methodology, Project administration, Writing – review & editing. LS-Z: Funding acquisition, Resources, Supervision, Writing – review & editing. FB: Software, Writing – review & editing. ZB: Investigation, Writing – review & editing. ZM: Investigation, Project administration, Writing – review & editing. IF: Writing - review and editing, Supervision. MR: Conceptualization, Data curation, Formal analysis, Funding acquisition, Investigation, Methodology, Project administration, Resources, Supervision, Validation, Visualization, Writing – original draft, Writing – review & editing.
